# Body image perception, smoking, alcohol use, indoor tanning, and disordered eating in young and middle-aged adults: findings from a large population-based Swedish study

**DOI:** 10.1186/s12889-021-10158-4

**Published:** 2021-01-12

**Authors:** Mattias Strand, Peeter Fredlund, Cecilia Boldemann, Anton Lager

**Affiliations:** 1 Centre for Psychiatry Research, Department of Clinical Neuroscience, Karolinska Institutet, & Stockholm Health Care Services, Stockholm County Council, 171 77 Stockholm, Sweden; 2 Department of Global Public Health, Karolinska Institutet, Stockholm, SE Sweden; 3 Centre for Epidemiology and Community Medicine, Stockholm County Council, Stockholm, SE Sweden

**Keywords:** Body image, Smoking, Indoor tanning, Alcohol, Public health, Epidemiology, Disordered eating, Stockholm public health cohort

## Abstract

**Background:**

Body image concerns may give rise to a multitude of risk behaviors, such as unhealthy weight control practices, disordered eating behaviors (e.g., compensatory purging or preoccupation with food), smoking, excessive alcohol intake, or sunbed use. However, the distribution of these risk behaviors across adulthood has rarely been studied.

**Methods:**

The aim of this study was to explore health risk behaviors as correlates of body image perception in a randomly selected study population of 30,245 individuals aged 30–66 in Stockholm, Sweden, utilizing data from the Stockholm Public Health Cohort. Bivariate correlations were explored and a hierarchic binary logistic regression analysis was performed.

**Results:**

The prevalence of body image discrepancy was higher among smokers and respondents displaying disordered eating behaviors. In contrast, alcohol and sunbed use were inversely correlated to body image discrepancy. Body mass index was the substantially strongest explanatory factor behind the observed variance in body image discrepancy, followed by loss-of-control eating. Notably, no major gender differences in body image perception were detected. Some unexpected patterns of association between variables other than body image perception, such as those between smoking, alcohol use, and sunbed use, were seen.

**Conclusions:**

Overall, the patterns differ substantially from what has previously been found in adolescent and young adult samples, indicating that the synergy and aggregation of risk behaviors observed among younger individuals may not apply to adults aged 30–66.

## Background

A discrepancy between perceived societal body ideals and one’s own experienced body shape is a risk factor for body dissatisfaction [1]—a divergence that in turn may impact individual health behaviors in destructive ways across the lifespan [1–7]. Unfortunately, the limited number of randomly selected samples as well as inconsistencies in operational definitions of body image perception and a lack of standardized cut-off points complicate the study of something as fundamental as the prevalence of body image concerns, not to mention the exploration of associated risk behaviors. A systematic review concluded that the prevalence of body dissatisfaction varies as widely as 11–72% for women and 8–61% for men [8]. Importantly, body image is a multidimensional construct that involves elements such as accuracy of perception, associated affects and emotions (negative or positive), and the centrality or salience of that appraisal in one’s cognitive schemata [9]—hence, it is not always clear what exactly is being measured in studies on body image concerns.

It has been suggested that body image concerns may give rise (or, at the very least, be correlated) to a multitude of risk behaviors, such as unhealthy weight control practices, disordered eating behaviors (e.g., compensatory purging behaviors or preoccupation with food), smoking, excessive alcohol intake, or sunbed use (including interrelationships between them) [8, 10–19]. Also, unhealthy weight control strategies and compensatory purging behaviors have been found to be significantly related to frequent indoor tanning among adolescent boys [15]. A common point of departure may be a negative body image which, apart from increasing the risk of unhealthy weight control behaviors, may fuel the aggregation of risk behaviors [10, 11]. Risk behaviors such as these are most commonly established at a young age [13, 14, 20]. This may, in turn, complicate preventive efforts in terms of information-based behavior change [21]. Yet, the distribution of these risk factors across adulthood has rarely been studied. The studies referenced above display a large heterogeneity in terms of study sample ages—there is often a focus on adolescents and young adults (employing various definitions), whereas less is known about body image perception in relation to risk behaviors among middle-aged adults.

Three of the aforementioned behaviors—smoking, excessive alcohol intake, and sunbed use—have been classified as group 1 carcinogens, i.e., they are proven carcinogenic [22]. Smoking and alcohol intake (at least beyond recommended maximum levels) additionally cause widespread ill-health and are major contributors to the global burden of disease [23]. Widespread morbidity caused by smoking, excessive alcohol intake, and sunbed use are to a great extent preventable as the risk factors can be avoided. In Sweden, the prevalence of smoking, although declining, is still 7% among both women and men [24]. The prevalence of alcohol use among Swedes has been stable over the past few years, at 77% [24]. However, the amounts of alcohol consumed have been reduced, as well as the prevalence of hazardous and binge drinking, especially among those aged 16–29. The data on sunbed use is somewhat conflicting. A report on environmental health concludes that the prevalence of indoor tanning in Sweden, at 11% among women and 6% among men in 2015, was halved between 2007 and 2015. A similar reduction, from 36 to 19%, was observed among those aged 18–29 years [25]. Other data, in contrast, indicates that no substantial change has occurred since 2007 [26, 27]. The above patterns should be viewed against a backdrop of bans on tobacco and alcoholic beverage sales to minors as well as prohibition for minors to use sunbeds, and mandatory information to be provided about associated health risks. Smoking is highly addictive, as is alcohol, particularly in established smokers [28]. Recent observations also indicate that exposure to ultraviolet radiation or ultraviolet-seeking behavior has addictive elements [29, 30]. In this context, it has been shown that self-perceived appearance, peer influence, and media images affect adolescents’ and young adults’ indoor tanning habits and that aspects of body image perception are linked not only to sunbed use but also to unhealthy practices of weight control [10, 31, 32].

The aim of this study was to explore disordered eating, smoking, alcohol intake, and sunbed use as “addictive correlates” of body image perception in a randomly selected study population drawn from the adult population of Stockholm County. A secondary aim was to explore the co-occurrence of these various health risk behaviors independent of body image perception in this age group. To the best of our knowledge, no studies have examined the integrated correlations of body image perception and the health risk behaviors outlined above in middle-aged populations. Since the trajectories and aggregation patterns of risk behaviors are likely to vary across the lifespan, it is of great significance to also study these characteristics in adults aged 30–66.

## Methods

### Data collection and study population

Data was retrieved from the Stockholm Public Health Cohort (SPHC), a population-based cohort study based on self-reported data via questionnaires [33]. Participants are 120,000 randomly selected adult residents of Stockholm County. Baseline surveys were conducted in 2002 and then re-conducted in 2006, 2010, and 2014, whereas new participants in the 2006 survey were followed up in 2010 and 2014. For the purpose of this cross-sectional study, respondents aged 30–66 years in the 2014 survey wave who provided valid data to the question of body image perception were included. This age span was chosen based on the facts that a) the question on indoor tanning in the questionnaire specifically asks about sunbed use before and up until the age of 30 (see below), which means that respondents younger than 30 years could not provide definitive answers, and b) indoor tanning was introduced in Sweden in 1978, which means that respondents older than 66 years were unlikely to have been exposed. Power calculations showed that an adequate statistical power for detecting relevant differences in body image perception could be anticipated utilizing these large-scale survey data. The study was approved by the Stockholm Public Health Cohort Steering Committee and the Regional Ethics Committee of Stockholm (#2016/2163–31).

### Study variables

In addition to questions concerning age, sex, weight, and height, the 2014 SPHC questionnaire included questions about body shape, smoking, alcohol intake, sunbed use, and disordered eating. Body mass index (BMI) was calculated using the respondents’ answers regarding weight and height. It should be noted that this study makes use of previously collected survey data and that it was therefore not possible to influence the choice of particular instruments and items included in the SPHC questionnaire.

#### Body image discrepancy

The nine-item figure rating scale (FRS), also known as the Stunkard scale [34], was used in two questions asking about body image: ”What body shape describes best your body frame today?” and “What body shape describes best how you would like to look?” The FRS is a visible measure of how an individual perceives his or her own physical appearance, where respondents are asked to choose among schematic body shape silhouettes. Even though the FRS has sometimes been seen as indicating body satisfaction/dissatisfaction, it appears more relevant to view it as a measure of body image perception and discrepancy, since it does not incorporate elements of affect and salience [9]. In this study, body image discrepancy was categorized in a dichotomized fashion, whereby respondents who displayed a ≤ 2-step deviation between experienced and desirable body shape were categorized as having no major body image discrepancy and respondents who displayed a > 2 step deviation were categorized as having a wish for being thinner. This categorization was based on the assumption that many respondents were likely to indicate a slight discrepancy (i.e., 1–2 steps) between experienced and desirable body shape without necessarily harboring any substantial body image concerns [9]. Respondents with a > 2 step deviation in the direction of wishing to be larger constituted only 0.2% of the material (*n* = 49) and were not included in the analysis.

#### Smoking

Two questionnaire items on smoking were used: 1) “Have you ever smoked on a more or less daily basis during at least 6 months?” (Yes/No) and 2) “Are you a current smoker?” (Yes/No).

#### Alcohol intake

Two items asked about drinking habits: 1) “What are your drinking habits like during an ordinary week?” (cross-table, days of the week and type of beverage: hard liquor 4 cl/dessert wine 5–8 cl/approx. 15 cl red or white wine/33 cl fortified cider or cooler/50 cl beer (quantities to be filled out per day)). 2) “How often during the last 12 months have you on one single occasion been drinking alcohol equaling 1 bottle of wine/4 cans of fortified beer/6 cans of standard beer?” (almost every day (> 5 days per week)/a couple of times per week (3–4 times per week)/occasionally during the week (1–2 times per week)/2–3 times per month/once per month/1–6 times per year/never). Hazardous drinking was defined as intake of > 17 g of 100% alcohol per day, in accordance with recent Swedish public health guidelines [35].

#### Indoor tanning

One item asked about sunbed use: “Have you ever tanned indoors before age 30?” (No/Yes, once /Yes, 2–10 times/Yes, more than 10 times).

#### Disordered eating

Parts of the SCOFF scale were employed. The SCOFF questionnaire [36] is composed of five questions on disordered eating, three of which (“Do you make yourself sick because you feel uncomfortably full?”, “Do you worry that you have lost control over how much you eat?”, and “Would you say that food dominates your life?”) are included in the 2014 wave of the SPHC. For the sake of brevity, these items will be referred to as compensatory vomiting, loss-of-control eating, and food dominates life, respectively. The SCOFF scale has demonstrated good validity as a screening tool for clinically relevant eating disorders [36].

### Statistical analysis

For the purpose of statistical analysis, age and BMI were treated as continuous variables whereas sex, body image concern, smoking, alcohol intake, and sunbed use were treated as dichotomous variables. For age and BMI in relation to body image concern, independent t-tests were performed. Contingency table analyses, including calculations of Cohen’s *d* effect sizes and odds ratios, were performed for body image concern data vs. data on smoking, alcohol intake, sunbed use, and disordered eating. Significant bivariate outcomes were then entered into a hierarchic binary logistic regression model, in which various predictor orders were tried until an optimal model fit in terms of explained variance as estimated by Nagelkerke R^2^ values was established. Finally, in order to provide an overall picture of associations within the data, contingency table analyses were performed for all dependent variables in relation to each other. In all analyses, an *α* level of < .05 was considered statistically significant. For all statistical analyses SPSS™ (Statistical Package for the Social Sciences for Windows, 26, SPSS Inc., Chicago IL) was used.

## Results

For this cross-sectional study, 30,245 respondents aged 30–66 provided valid data on the survey questions of body image. Women constituted 59.0% (*n* = 17,834) of the sample, whereas 41.0% (*n* = 12,411) were men. The mean age among all respondents was 50.5 years (median 51.0, standard deviation (SD) 10.0). Among women and men, the mean age was 50.1 years (median 50.0, SD 10.0) and 51.1 years (median 52.0, SD 9.9), respectively. The mean BMI among all respondents was 25.3 kg/m^2^ (median 24.7, SD 4.1, minimum value 13.3, maximum value 58.8). Among women and men, the mean BMI was 24.7 kg/m^2^ (median 23.9, SD4.3) and 26.2 kg/m^2^ (median 25.6, SD 3.7), respectively. Further descriptive data for the cohort is provided in Table 1. Of all respondents, 94.3% (*n* = 28,513) indicated a ≤ 2-step deviation on the FRS, i.e., no major body image discrepancy. The largest share, 42.8% (*n* = 12,932), displayed a 1-step deviation in the direction of wishing to be thinner, whereas 30.3% (*n* = 9157) displayed no deviation and 17.7% (*n* = 5358) displayed a 2-step deviation in the direction of wishing to be thinner. In contrast, only 5.6% (*n* = 1683) indicated substantial body image discrepancy, i.e., a > 2 step deviation on the FRS. Only 0.2% (*n* = 49) indicated a > 2 step deviation in the direction of wishing to be larger.

**Table 1:** Descriptive data for the Stockholm Public Health Cohort 2014 wave

	***n*** (%)	
	Yes	No
**Ever smoked > 6 months**		
All	12,285 (40.8)	17,816 (59.2)
Women	7513 (42.3)	10,231 (57.7)
Men	4772 (38.6)	7585 (61.4)
**Current smoker**		
All	2462 (8.2)	27,625 (91.8)
Women	1558 (8.8)	16,178 (91.2)
Men	904 (7.3)	11,447 (92.7)
**Alcohol last 12 months**		
All	27,293 (90.9)	2747 (9.1)
Women	15,840 (89.5)	1862 (10.5)
Men	11,453 (92.8)	885 (7.2)
**Hazardous drinking**		
All	7969 (26.4)	22,246 (73.6)
Women	2973 (16.7)	14,842 (83.3)
Men	4996 (40.3)	7404 (59.7)
**Sunbed use**		
All	17,821 (59.2)	12,289 (40.8)
Women	12,242 (68.9)	5514 (31.1)
Men	5579 (45.2)	6775 (54.8)
**Compensatory vomiting**		
All	378 (1.3)	29,658 (98.7)
Women	264 (1.5)	17,461 (98.5)
Men	114 (0.9)	12,197 (99.1)
**Loss-of-control eating**		
All	2311 (7.7)	27,677 (92.3)
Women	1673 (9.5)	16,007 (90.5)
Men	638 (5.2)	11,670 (94.8)
**Food dominates life**		
All	1582 (5.3)	28,334 (94.7)
Women	1142 (6.5)	16,514 (93.5)
Men	440 (3.6)	11,820 (96.4)

Missing data was in the range of 0.1–1.8% of all respondents for the various items and is not displayed

Significant differences in BMI were seen in the two body image perception groups, with a higher BMI being associated with a higher prevalence of body image discrepancy among both men and women (see Table 2). The effect sizes for these differences were very large, indicating differences larger than 1 SD between the means. A higher mean age was also significantly associated with a higher prevalence of body image discrepancy in the group as a whole and in men, but not in women, although these effect sizes where very small.

**Table 2:** Associations between body image discrepancy, age, and body mass index

	Body image discrepancy			No major body image discrepancy				
	***n***	Mean	SD	***n***	Mean	SD	***p***	Cohen’s ***d***
**Age (years)**								
All	1683	51.5	9.8	28,513	50.5	10.0	<.001	0.10
Women	986	50.5	9.8	16,838	50.1	10.0	.151	0.04
Men	697	52.8	9.6	11,675	51.1	9.9	<.001	0.17
**BMI (kg/m**^**2**^**)**								
All	1646	31.3	5.4	28,005	25.0	3.8	<.001	1.35
Women	964	31.6	5.6	16,502	24.3	3.9	<.001	1.52
Men	682	30.9	5.0	11,503	25.9	3.4	<.001	1.17

*SD* Standard deviation

The prevalence of body image discrepancy did not differ significantly between men and women (see Table 3). Being an ever-smoker and/or current smoker was significantly associated with an increased prevalence of body image discrepancy, as were all three SCOFF items (compensatory vomiting, loss-of-control eating, and experiencing food as dominating one’s life). In contrast, alcohol use and sunbed use (the latter in the full cohort and among men but not among women) were inversely correlated to body image discrepancy. No significant associations were seen regarding body image discrepancy, hazardous drinking, and frequent sunbed use.

**Table 3:** Associations between body image discrepancy and health risk behaviors

	All				Women				Men			
	***n*** (%)				***n*** (%)				***n*** (%)			
	Body image discrepancy	No major body image discrepancy	***χ2***	OR (95% CI)	Body image discrepancy	No major body image discrepancy	***χ2***	OR (95% CI)	Body image discrepancy	No major body image discrepancy	***χ2***	OR (95% CI)
**Sex**			0.144									
Men	697 (5.6)	11,675 (94.4)		1.02 (0.92–1.13)								
Women	986 (5.5)	16,838 (94.5)										
**Ever smoked > 6 months**			84.933				45.273				40.433	
Yes	863 (7.0)	11,397 (93.0)		**1.59 (1.44–1.75)**	516 (6.9)	6991 (93.1)		**1.55 (1.37–1.77)**	347 (7.3)	4406 (92.7)		**1.64 (1.41–1.91)**
No	811 (4.6)	16,981 (95.4)			464 (4.5)	9763 (95.5)			347 (4.6)	7218 (95.4)		
**Current smoker**			37.261				26.474				11.244	
Yes	203 (8.3)	3420 (92.6)		**1.60 (1.38–1.87)**	130 (8.4)	1426 (91.6)		**1.65 (1.36–2.00)**	73 (8.1)	826 (91.9)		**1.54 (1.19–1.98)**
No	1468 (5.3)	26,115 (94.7)			847 (5.2)	15,323 (94.8)			621 (5.4)	10,792 (94.6)		
**Alcohol last 12 months**			73.933				58.139				17.661	
Yes	1415 (5.2)	25,841 (94.8)		**0.54 (0.47–0.63)**	799 (5.0)	15,034 (95.0)		**0.52 (0.44–0.62)**	616 (5.4)	10,807 (94.6)		**0.59 (0.46–0.76)**
No	250 (9.1)	2485 (90.9)			173 (9.3)	1686 (90.7)			77 (8.8)	799 (91.2)		
**Hazardous drinking**			1.949				3.407				0.023	
Yes	467 (5.9)	7493 (94.1)		1.08 (0.97–1.21)	185 (6.2)	2786 (93.8)		1.17 (0.99–1.38)	282 (5.7)	4707 (94.3)		1.01 (0.87–1.18)
No	1210 (5.4)	20,996 (94.6)			798 (5.4)	14,036 (94.6)			412 (5.6)	6960 (94.4)		
**Sunbed use**			7.150				1.992				5.994	
Yes	939 (5.3)	16,682 (94.7)		**0.87 (0.79–0.97)**	658 (5.4)	11,580 (94.6)		0.91 (0.79–1.04)	281 (5.1)	5282 (94.9)		**0.82 (0.70–0.96)**
No	735 (6.0)	11,526 (94.0)			325 (5.9)	5183 (94.1)			410 (6.1)	6343 (93.9)		
**Sunbed use > 10 times**			0.495				0.000				2.044	
Yes	554 (5.7)	9160 (94.3)		1.04 (0.94–1.15)	405 (5.5)	6911 (94.5)		1.00 (0.88–1.14)	149 (6.2)	2249 (93.8)		1.15 (0.95–1.38)
No	1120 (5.5)	19,228 (94.5)			578 (5.5)	9852 (94.5)			542 (5.5)	9376 (94.5)		
**Compensatory vomiting**			37.306				8.012				46.528	
Yes	48 (12.7)	329 (87.3)		**2.52 (1.85–3.42)**	25 (9.5)	239 (90.5)		**1.81 (1.19–2.75)**	23 (20.4)	90 (79.6)		**4.40 (2.76–7.00)**
No	1621 (5.5)	27,989 (94.5)			953 (5.5)	16,498 (94.5)			668 (5.5)	11,491 (94.5)		
**Loss-of-control eating**			1320.324				876.967				460.122	
Yes	513 (22.2)	1796 (77.8)		**6.60 (5.85–7.36)**	356 (21.3)	1316 (78.7)		**6.69 (5.80–7.71)**	157 (24.6)	480 (75.4)		**6.84 (5.60–8.35)**
No	1153 (4.2)	26,477 (95.8)			622 (3.9)	15,376 (96.1)			531 (4.6)	11,101 (95.4)		
**Food dominates life**			615.559				452.688				168.367	
Yes	307 (19.4)	1273 (80.6)		**4.82 (4.21–5.53)**	221 (19.4)	920 (80.6)		**5.06 (4.29–5.96)**	86 (19.6)	353 (80.4)		**4.55 (3.54–5.84)**
No	1347 (4.8)	26,942 (95.2)			748 (4.5)	15,757 (95.5)			599 (5.1)	11,185 (94.9)		

Significant findings (i.e., the 95% confidence interval (CI) does not overlap 1) are marked in bold. *OR* Odds ratio

Based on the bivariate analyses described above, the variables significantly associated with body image discrepancy—i.e., BMI, the three SCOFF items, being an ever-smoker, being a current smoker, alcohol use, and sunbed use —were chosen for inclusion in the hierarchic binary logistic regression model. This model showed that 25.3% of the variance in body image discrepancy was explained by BMI alone (Nagelkerke R^2^ = 0.253). Adding loss-of-control eating to BMI modestly increased the explained variance to 28.1% (Nagelkerke R^2^ = 0.281). The addition of further risk behaviors to the model merely increased the explained variance to 28.9% (Nagelkerke R^2^ = 0.289) in the final block. Notably, the presence of compensatory vomiting or sunbed use did not significantly improve the regression model fit.

Significant associations were observed between alcohol use and/or hazardous drinking and being an ever-smoker as well as between hazardous drinking and being a current smoker in both women and men (see Table 4). In contrast, an inverse association was observed between alcohol use and being a current smoker. Furthermore, ever-smokers and current smokers were less likely to have used a sunbed or to have been frequent sunbed users, whereas alcohol use was associated with sunbed use among both men and women. Compensatory vomiting was associated with current smoking among both men and women. All three SCOFF items were inversely associated with alcohol use. Among women, loss-of-control eating was associated with sunbed use and frequent sunbed use, and experiencing food as dominating one’s life was associated with frequent sunbed use. The three SCOFF items displayed a strong internal association.

**Table 4:** Association matrix of health risk behaviors presented as pairwise odds ratios with 95% confidence intervals in parentheses

		Alcohol last 12 m	Hazardous drinking	Sunbed use	Sunbed use > 10 times	Compensatory vomiting	Loss-of-control eating	Food dominates life
**Ever smoked > 6 m**	Women	**1.72 (1.55–1.90)**	**2.63 (2.42–2.85)**	**0.87 (0.82–0.93)**	1.01 (0.95–1.07)	1.24 (0.98–1.59)	1.03 (0.93–1.14)	1.03 (0.91–1.16)
	Men	**1.05 (0.91–1.21)**	**2.07 (1.96–2.23)**	**0.67 (0.62–0.72)**	**0.73 (0.66–0.80)**	1.09 (0.75–1.59)	1.16 (0.99–1.37)	1.14 (0.94–1.38)
**Current smoker**	Women	**0.84 (0.72–0.99)**	**1.99 (1.77–2.25)**	**0.73 (0.66–0.81)**	**0.78 (0.70–0.87)**	**1.71 (1.21–2.44)**	0.98 (0.82–1.17)	**0.76 (0.60–0.96)**
	Men	**0.60 (0.48–0.75)**	**1.35 (1.18–1.54)**	**0.57 (0.49–0.66)**	**0.62 (0.51–0.75)**	**2.29 (1.36–3.86)**	1.04 (0.77–1.41)	1.37 (0.99–1.89)
**Alcohol last 12 m**	Women		n/a	**2.86 (2.59–3.15)**	**2.34 (2.10–2.62)**	**0.38 (0.28–0.50)**	**0.59 (0.51–0.68)**	**0.65 (0.55–0.78)**
	Men		n/a	**2.21 (1.90–2.57)**	**2.03 (1.63–2.51)**	**0.22 (0.14–0.34)**	**0.46 (0.36–0.58)**	**0.42 (0.32–0.55)**
**Hazardous drinking**	Women			0.96 (0.88–1.04)	0.98 (0.91–1.07)	0.95 (0.68–1.32)	0.92 (0.80–1.06)	1.02 (0.87–1.20)
	Men			1.06 (0.99–1.14)	1.04 (0.95–1.14)	0.80 (0.54–1.17)	**0.73 (0.61–0.86)**	0.96 (0.79–1.16)
**Sunbed use**	Women				n/a	0.80 (0.62–1.03)	**1.16 (1.04–1.30)**	1.07 (0.94–1.23)
	Men				n/a	0.86 (0.59–1.26)	0.96 (0.82–1.13)	0.97 (0.80–1.18)
**Sunbed use > 10 times**	Women					1.08 (0.85–1.38)	**1.33 (1.20–1.47)**	**1.14 (1.01–1.29)**
	Men					1.18 (0.75–1.84)	1.05 (0.86–1.28)	0.91 (0.71–1.16)
**Compensatory vomiting**	Women						**7.93 (6.18–10.17)**	**5.65 (4.27–7.47)**
	Men						**14.79 (10.08–21.69)**	**7.96 (5.05–12.54)**
**Loss-of-control eating**	Women							**22.63 (19.78–25.90)**
	Men							**16.87 (13.63–20.88)**

Significant findings (i.e., the 95% confidence interval does not overlap 1) are marked in bold

## Discussion

The findings of the present study reveal that among 30,245 randomly selected residents of Stockholm County aged 30–66, body image discrepancy (i.e., wish for thinness) was associated with a higher BMI and was also more prevalent among ever-smokers and/or a current smokers and among respondents displaying disordered eating behaviors (i.e., compensatory vomiting, loss-of-control eating, or experiencing food as dominating one’s life). In contrast, alcohol use and sunbed use were inversely correlated to body image discrepancy. In a hierarchical binary logistic regression model including these variables, BMI was the one variable with the substantially strongest influence on the observed variance in body image discrepancy, followed by experiencing loss-of-control eating. The rest of the included variables only slightly improved the model fit.

One of the most intriguing facets of these patterns among young and middle-aged adults, discussed in more detail below, is the fact that they differ substantially from what has previously been found in adolescent and young adult samples. The synergy and aggregation of risk behaviors typically seen among younger individuals were not observed here. Notably, body image discrepancy did not seem to contribute to an aggregation of risk behaviors—on the contrary, a number of risk behaviors (such as alcohol use, hazardous drinking, and sunbed use) were less prevalent among those young and middle-aged adults who displayed a discrepancy in body image perception.

Factors such as thin and/or athletic body ideals [31, 37], weight stigma [38], and concern over potential medical risks associated with overweight and obesity [39–41] are likely to make respondents with high BMIs more concerned about their own body shape. The strong association observed here between body image discrepancy and a higher BMI may therefore be expected; yet, only 5.6% of respondents indicated a discrepancy of > 2 steps on the FRS between perceived and ideal body shape in the direction of wishing to be thinner, indicating that the vast majority of respondents were fairly satisfied with their body shape. This relatively small number also implies that the detected body image discrepancy does not simply represent a more general wish for a youthful body in adults aged 30–66. At the same time, very few—only 0.16% of respondents—displayed a > 2 steps discrepancy in the direction of wishing to be larger; this small number is surprising, considering such phenomena as muscle dysmorphia and that athletic body ideals may also very well make people wish to be larger [15, 42]. The fact that the FRS displays larger body shapes as rotund rather than muscular may contribute to this low number of respondents indicating a wish to be larger.

Furthermore, a substantial association between disordered eating behaviors and body image discrepancy was observed. Indeed, body image discrepancy is often, although not always, a driving force in dieting and an established risk factor for the development of restrictive eating disorders [31, 32]. Likewise, individuals with bulimia nervosa or binge eating disorder typically experience body image discrepancy as a core element of their suffering [43]. Naturally, the subjective presence of disordered eating behaviors does not mean that respondents necessarily fulfil diagnostic criteria for an eating disorder, although the SCOFF scale is commonly used as a screening tool in the assessment of eating disorders. Also, the observed association between body image discrepancy and disordered eating behaviors does not imply that the impact of body image concerns on eating behaviors is unidirectional. Patterns of disordered eating may also strengthen and perpetuate body image discrepancy—indeed, targeting disordered eating behaviors rather than body image concerns is usually the first step in the clinical treatment of eating disorders, partly based on the observation that reducing eating disorder behaviors tend to also diminish the strength of body image concerns [44]. However, it is also noteworthy that a large majority of the respondents who displayed disordered eating behaviors did not indicate a substantial wish for being thinner. This raises questions about the nature of subclinical disordered eating and its relationship with body image—in fact, a substantial share of those who reported disordered eating may relate these behaviors to issues such as sensory selectivity, appetite dysregulation, or fear of underlying medical issues rather than body image concerns.

The fact that ever-smokers as well as current smokers displayed a higher prevalence of body image discrepancy could hypothetically be due to the fact that smoking may be used by some people as a means of weight control [12, 45]. Similarly, the observed inverse correlation between alcohol use and body image concerns—i.e., respondents who reported no alcohol intake in the past 12 months displayed a higher prevalence of body image discrepancy—may in part express a fear of weight gain due to the high calorie content of alcoholic beverages [46]. Even though an association between body dissatisfaction and binge drinking has also been found in young adults [47], no such pattern was observed in our young and middle-aged adult study population. The same conclusion may be drawn regarding the observed inverse association between all disordered eating behaviors and alcohol use, which may be due to concerns about excessive caloric intake. Among individuals with an eating disorder, alcohol use is significantly more common in those with binge-purge behaviors [48], possibly related to associated impairments in impulse control in the same group [49]. In our study population, however, those who displayed compensatory vomiting were also less likely to use alcohol. This may reflect different patterns of aggregated risk behaviors among adults aged 30–66.

Since people who engage in indoor tanning mostly do so for cosmetic reasons [10], a positive correlation between sunbed use and body image discrepancy might be expected. The contrasting finding of lower prevalence of body image discrepancy among those who had engaged in sunbed use in the present study could, however, indicate that substantial body image concerns are related to avoiding situations where one’s undressed body is on display in places such as locker rooms, gyms etc. and associated feelings of shame [50]. Notably, body shame among people with overweight or obesity is also reported to be a contributing barrier to physical exercise, such as jogging outdoors [38]. However, for the same reason, others have hypothesized that individuals with a higher BMI may in fact be more likely to use indoor tanning facilities rather than sunbathing outdoors [51]. Moreover, some adolescents who are classified as overweight may believe that tanning makes them appear more slim [51]. Regarding disordered eating and sunbed use, compensatory purging behaviors such as vomiting have been associated with indoor tanning in adolescents [10]. Among our adult respondents, we found no such association, although loss-of-control eating was associated with sunbed use and frequent sunbed use, and experiencing food as dominating one’s life was associated with frequent sunbed use among women.

Some surprising patterns of associations between variables other than body image discrepancy, such as those between smoking, alcohol use, and sunbed use, were seen in this study population. Previous studies, predominantly carried out among adolescents and young adults, have shown strong relationships between certain risk behaviors, e.g., sunbed use and smoking [16] or disordered eating [13], indicating inherent synergetic dynamics in the aggregation of risk behaviors. These observations did not apply to the adults aged 30–66 in this study. Here, for example, smokers and those with body image discrepancy tended to use sunbeds less. Overall, body image discrepancy did not seem to contribute to an aggregation of risk behaviors—on the contrary, some risk behaviors were less prevalent among respondents who displayed a discrepancy in body image perception (e.g., alcohol use and sunbed use). Thus, much of the aggregation of risk behaviors found among adolescents and young adults did not apply to the adults aged 30–66 in the present study. Regarding sunbed use, it is uncertain how many of respondents that still used sunbeds when the data used in this study was collected. Only exposure before age 30 is known—this, however, entails a doubled risk of skin cancer [52]. A fair share of the study population may possibly have given up indoor tanning at the time of data collection. As individuals grow older, develop more mature relationships, start a family, strive for a professional career, etc., acting upon concerns about appearances typically becomes less important [53, 54] even if striving for thinness or athleticism can certainly continue to be an important ideal well into middle age [55]. This, combined with realistic weight concerns emanating from medical risks associated with obesity, may further attenuate the links between risk behaviors and body image perception among young and middle-aged adults. Even so, the strong association between smoking and sunbed use remains, indicating that some aggregation of risk behaviors exists irrespective of age, although it does not necessarily involve body image perception among adults aged 30–66. Future studies may further elucidate the strong relationship between smoking and sunbed use and its implications for other risk behaviors.

### Strengths and limitations

The SPHC is one of the largest available public health cohorts of its kind, allowing for statistically robust analysis of health-related behaviors. In the present study, merely 2.7% of respondents aged 30–66 were excluded based on not having provided valid data on body image perception, resulting in a cohort of 30,245 respondents. As seen in Table 1, missing data for dependent variables was negligible throughout. The survey data in the SPHC has been complemented by information from Swedish longitudinal health and sociodemographic data registries, which is generally considered as being of very high quality in terms of scope and reliability [33, 56].

A number of potential limitations should also be mentioned. For example, our body image discrepancy construct—i.e., utilizing a 2-step deviation between perceived and ideal body shape as cutoff—allows us to grasp the direction and size of discrepancy in respondents’ body image discrepancy but does not tell us anything about what affect and salience they assign to it. For example, it could very well be that some respondents that indicated that they wish to be > 2 steps thinner on the FRS would indeed change their body shape if they were somehow offered a “magic” opportunity to do so (e.g., to lose weight without dispensing with tasty food rich in calories), but that regardless of this it is not all that important to them and they do not spend much time worrying about their body shape in everyday life. Moreover, choosing another arbitrary cutoff value, such as > 1 step deviation (which would render 23.3% of respondents dissatisfied with their body shape), could potentially alter the outcome. However, the fairly small percentage of respondents that report body image discrepancy when utilizing the current construct indicates that it does indeed capture those with substantial concern.

In administering a public health survey, there is an inherent trade-off between the number of useful instruments to include and the “workload” tolerated by respondents. This study relies on the measures and items already available in the SPHC. The inclusion of, for example, all five SCOFF items and body image measures complementary to the FRS may naturally have been preferable, but this was beyond our control at the time of this study.

Naturally, the presence of unmeasured confounders that impact our findings cannot be ruled out. Moreover, the cross-sectional study design does not allow for the analysis of causal effects, possible dynamics such as triggers, aggregation, and synergies of risk behaviors, or the workings of addiction and other consequences over time [29]. For example, any conclusions regarding to what extent the risk behaviors investigated in this study were established during adolescence or young adulthood cannot be made. In any case, our findings indicate that the strong aggregation of risk behaviors seems to diminish with age.

### Clinical and research implications

The discrepant patterns of risk behavior aggregation observed in adults aged 30–66 have potential implications for prevention and clinical assessment. For example, if risk behaviors such as alcohol use, hazardous drinking, and sunbed use are actually less prevalent among those young and middle-aged adults who display a discrepancy in body image perception, preventive measures as well as treatment interventions may need to be individually tailored according to different assumptions about risk aggregation than those typically employed for younger people. Moreover, the fact that a large majority of those respondents who displayed disordered eating behaviors did not indicate a substantial body image discrepancy implies that factors other than wish for thinness should be considered in relation to disordered eating in young and middle-aged adults. For example, in this age group, health anxiety—of a realistic or unrealistic nature—may possibly have more impact on preoccupation with food [57].

Importantly, relatively little is known about the trajectories of risk behaviors related to body image across the lifespan. This study indicates that patterns of synergy and aggregation of risk behaviors among adults aged 30–66 in Stockholm county differ substantially from what has previously been found in adolescent and younger adult samples. These findings need to be further explored and corroborated in other settings, preferably with the use of more detailed and nuanced measures of body image concerns.

## Conclusion

Among randomly selected residents of Stockholm County aged 30–66, the prevalence of body image discrepancy was observed to be positively correlated to BMI and was also higher among smokers (current or former) and among respondents displaying disordered eating behaviors. In contrast, alcohol use and sunbed use were inversely correlated to body image discrepancy. Logistic regression indicated that BMI was the one variable with the substantially strongest influence on the observed variance in body image discrepancy, followed by the presence of loss-of-control eating. Notably, no major gender differences in body image discrepancy were detected. Moreover, some unexpected patterns of association between variables other than body image perception, such as those between smoking, alcohol use, and sunbed use, were seen, indicating that much of the aggregation of risk behaviors observed among adolescents and younger adults in previous studies may not apply to adults in older age spans.

## Data Availability

The anonymized datasets used and analyzed during the current study are available from the corresponding author on reasonable request.

## Abbreviations

BMI: Body mass index
FRS: Figure Rating Scale
SPHC: Stockholm Public Health Cohort
